# Physical activity and risk behaviors among adolescents: the mediating roles of diet, sleep, mental health, and substance use

**DOI:** 10.3389/fpubh.2025.1665023

**Published:** 2025-10-02

**Authors:** Feng Tian, Fengbo Liu

**Affiliations:** College of Physical Education, Zhengzhou University of Light Industry, Zhengzhou, China

**Keywords:** physical activity, suicide risk, substance use risk, behavior mechanism, emotional mechanisms

## Abstract

**Background:**

Suicide risk (SR), including suicidal ideation and suicide attempts, is a critical public health issue, particularly among adolescents. Emerging evidence highlights substance use risk (SUR) as a prominent factor elevating SR among youth. Physical activity (PA) has demonstrated protective effects against both SR and SUR, potentially due to its broad benefits for physical and mental well-being. Despite accumulating evidence supporting these associations, the underlying mediating mechanisms remain poorly understood, especially the roles of sleep duration (SD), healthy diet (HD), and mental health (MH). Clarifying the complex interactions and sequential mediating pathways among these variables is essential for developing effective interventions to mitigate SR and SUR among adolescents.

**Methods:**

We analyzed data from the 2023 U.S. Youth Risk Behavior Surveillance System (YRBSS), including 15,734 adolescents after excluding cases with missing key variables. PA, HD, SUR, SR, SD, and MH were measured using validated YRBSS items, with multi-item constructs modeled as latent variables. Structural equation modeling and bootstrapped mediation analyses (5,000 resamples) were conducted in R 4.5, with model fit evaluated using RMSEA (< 0.08) and CFI/TLI (> 0.95).

**Results:**

Higher PA significantly predicted lower SR (*β* = −0.024, *p* < 0.001) and SUR (*β* = −0.021, *p* < 0.001). Notably, direct pathways from PA to SR and SUR revealed small positive effects, indicating suppression effects and emphasizing the importance of indirect mechanisms. MH emerged as a crucial mediator between PA and both SUR and SR. Additionally, SD significantly mediated the association between PA and SUR. Chain mediation analyses revealed significant sequential pathways: PA → SD → MH → SUR; PA → MH → SUR → SR; and PA → HD → MH → SR, highlighting complex protective mechanisms linking physical activity with adolescent health outcomes. Conversely, HD alone did not significantly mediate the PA–SR relationship, and the PA → SD → SUR → SR pathway was not supported.

**Discussion:**

The beneficial effects of PA on reducing SR and SUR among adolescents primarily operate indirectly through enhanced MH and SD, with minor contributions from dietary habits. These findings underscore the importance of integrated intervention strategies targeting MH and sleep quality to maximize the protective benefits of PA for adolescents.

## 1 Introduction

Suicide risk (SR), encompassing both suicidal ideation and suicide attempts, represents a major public health concern (1). Although suicide is not classified as a disease, the World Health Organization (WHO) has reported that approximately 727,000 people die by suicide each year. Among individuals aged 15–29 years, suicide was the third leading cause of death overall, ranking second among females and third among males (2). Notably, WHO has indicated that over half of individuals who die by suicide are under 50 years old. In a given year, suicide accounts for approximately 1.1% of deaths worldwide, however, the fatality rate is significantly higher among individuals aged 15–29 years. From a practical perspective, each suicide death represents a personal tragedy and is estimated to indirectly affect numerous individuals, including family members, friends and the broader community (3).

These data highlight the importance of SR intervention, particularly among adolescents. Advances in our understanding of SR assessment and intervention have contributed to reductions in both SR and suicidal behavior. Researchers have identified several predictive factors associated with suicidal behavior and have compared their relative importance. From a psychiatric perspective, SR has been conceptualized as the result of three interrelated factors: perceived burdensomeness, thwarted belongingness, and acquired capability for suicide, these factors may interact to produce a heightened risk of suicide behavior (4). It is worth noting that over 90% of suicide cases involve at least one mental disorder, with major depressive disorder, substance use disorder, and anxiety disorders being the most common (5). From a comprehensive perspective, major contributing factors to SR include psychiatric disorders, childhood trauma, social deprivation, and neurobiological mechanisms (6). For adolescents, several specific risk factors have been identified, including family conflict, academic pressure, peer relationships difficulties, and mental disorders (7). It is worth noting that substance use risk (SUR) is directly associated with SR, and has gradually become a critical focus in to SR research.

SUR among adolescents encompasses behaviors of high-risk substance use, such as misuse of prescription medications, illicit drug experimentation, or injection drug use, which carry elevated potential for adverse outcomes including injury, legal consequences, dropout, or even death (8). Importantly, The Diagnostic and Statistical Manual of Mental Disorders (DSM-5) defines substance use disorder (SUD) as a chronic condition characterized by impaired control over substance use and continued use despite significant harm. Adolescents, whose brains are still in a critical developmental stage, are particularly vulnerable to such risks (9). Individuals with substance use behaviors are approximately three times more likely to experience elevated SR compared to those without such behaviors (10). Alcohol-related disorders significantly increase suicidal ideation, suicide attempt, and completed suicide (11). One investigation reported that approximately 25–33% of adolescents involved in suicide cases had a history of alcohol or other substance use. Moreover, the risk of substance use increases by approximately 30% even after a single episode of alcohol consumption (12). Moreover, a significant relationship has been observed between substance use disorder and SR among youth (13). In addition to alcohol consumption, the use of other illicit substances such as heroin and cocaine is also significantly associated with SR (14). A direct study indicated that individuals with substance us disorders significantly predicted SR, with odds ratios (ORs) ranging from 2.0 to 11.2 (15). According to the DSM-5, both SR and SUR are considered manifestations of mental behavior, or neurodevelopmental disorders (9). These risks are often accompanied by three key characteristics: tolerance, withdrawal, and compulsive use behavior, all of which are indicative of a chronic condition (16). According to the ICD-11, SUR is categorized into three stages of harm: single episodes of harmful use, harmful pattern of use, and substance dependence. These stages contribute to serious and long-term harm across physical, psychological, and social domains (17). We need to attend to these growing longer-term mental and complex consequences of SR and SUR. Identifying effective strategies to intervene in SR and SUR is essential for public health.

Insufficient physical activity (PA) has been identified as a potential risk factor for numerous serious conditions, including cardiovascular disease (18), cancer (19), osteoporosis (20), diabetes, obesity, hypertension (21), anxiety, and depression (22). Regular PA is an essential and effective intervention for a wide range of chronic conditions, and a linear relationship has been observed between PA and mental health (23). Because of PA can effectively promote overall well-being, it provides significant benefits for both physical and mental health (24). Enhancing PA levels has been regarded as a critical strategy for improving overall health (25).

Adolescent substance use is embedded within broader psychosocial systems. Problem Behavior Theory posits that adolescent health-compromising behaviors, such as substance use, often cluster within the same behavioral syndrome, whereas protective factors like sport involvement and PA can buffer these risks (26). The contemporary suicide models such as the Interpersonal Theory of Suicide and the Integrated Motivational–Volitional (IMV) model highlight the role of belongingness, emotional regulation, and self-control—factors that can be enhanced through PA—in reducing SR (4, 27). PA appears to have the potential to reduce SR and SUR due to its beneficial properties. Current research has indicated a significant relationship between PA, SR, and SUR. For example, A comprehensive review demonstrated that higher PA levels can significantly predict lower suicidal ideation (OR = 0.91, 95% CI [0.51, 0.99]) (28). A meta-analysis of 17 randomized controlled trials (RCTs) found that exercise interventions significantly reduced suicide attempts compared to inactive control groups (OR = 0.23, 95% CI [0.09, 0.67]) (29). However, the underlying mechanisms of these complex relationships remain insufficiently understood, particularly among adolescents, who are more likely to contribute key factors to associated with these risks. Therefore, we aim to further clarify the complex relationship between PA, SR, and SUR, with particular attention to identifying the significant mediators involved.

In literature, the term exercise is often used to distinguish it from general PA, as PA includes both structured, scheduled exercise and unstructured daily activities such as household chores (30). Rather than focusing on whether PA is classified as structured exercise or part of daily routines, greater emphasis should be placed on its total volume, frequency, and intensity (31). ACSM (American College of Sports Medicine) defines PA as any bodily movement produced by skeletal muscles that results in energy expenditure, and exercise is defined as a planned, structured, and repetitive form of physical activity intended to improve or maintain one or more components of physical fitness (32). In this study, we do not strictly differentiate PA and exercise, instead, PA is broadly defined as any activity that results in energy expenditure.

### 1.1 The direct effects of PA on SR and SUR

Previous studies have demonstrated the benefits of PA across various age groups and populations. Among preschool children, even a single bout of moderate-to-vigorous PA has been shown to improve sleep quality, enhance cognitive function, and regulate insulin sensitivity, and regular PA is associated with more pronounced improvements in these domains (33). Moreover, individuals across diverse backgrounds, including pregnant and postpartum women, people of all genders and ages, individuals with disabilities, and those seeking to reduce their risk of disease, can benefit from PA interventions (34). A substantial body of epidemiological research has demonstrated that PA can significantly enhance self-esteem and subjective well-being. Adults who engage in regular PA tend to report fewer symptoms of depression and anxiety. These findings support the view that exercise may play a preventive role in the development of mental disorders (35). The theoretical research of PA suggests that it can reduce body fat, which subsequently enhances cardiopulmonary function, improves muscular strength, and promotes skeletal health, ultimately contributing to better mental health (36). We have discussed the psychological definitions of SR and SUR, both of which can lead to serious consequences. However, accumulating evidence suggests that these risks may be mitigated through PA. Based on these findings, we hypothesize that there may be a significant relationship between PA, SR, and SUR among adolescents. Accordingly, we propose the following research question:

H2a: High levels of PA will significantly predict lower levels of SR.

H2b: High levels of PA will significantly predict lower levels of SUR.

### 1.2 Mediating roles of sleep, diet, and mental health

The complex mediating roles of sleep duration (SD), healthy diet (HD), and mental health (MH) in the associations between PA, SR, and SUR among adolescents warrant further examination. Although direct significant relationships have been identified among SD, HD, MH, SR and SUR, research specifically focusing on adolescents remains limited. For example, a meta-analysis has identified that insomnia can prospectively predict suicidal thoughts and behaviors (37). Sleep disturbances can also be an important risk factor for SR and suicide attempts among otherwise healthy adolescents (38). A healthy diet, such as the Mediterranean diet, may prospectively reduce depressive symptoms, and depression is one of the key antecedent factors associated with increased suicide risk (39). Moreover, individuals with depression are estimated to have a 20-fold increased risk of suicide compared to the general population. Depression is currently recognized as one of the most well-established and stable predictors of suicide (40). Depression and SUR are highly comorbid and exert bidirectional influences. On one hand, depression may lead to substance misuse through a self-medication mechanism; on the other hand, SUR can exacerbate depressive symptoms and increase the risk of suicide (41). Individuals with SUR exhibit significantly elevated suicide risk, with ORs typically ranging from 2.5 to 7.5. The risk is particularly pronounced among individuals with polysubstance use, as well as those using alcohol and opioids (42). Given the established associations among PA, SR, and SUR, it is worth exploring whether SD, HD, and MH may serve as potential mediators in these relationships. Accordingly, we propose the following research question:

H1a: SD significantly mediates the relationship between PA and SUR.

H1b: MH significantly mediates the relationship among PA, SUR and SR.

H1c: HD significantly mediates the relationship between PA and SR.

### 1.3 Chain mediation models linking PA to SR and SUR

**Figure 1:**
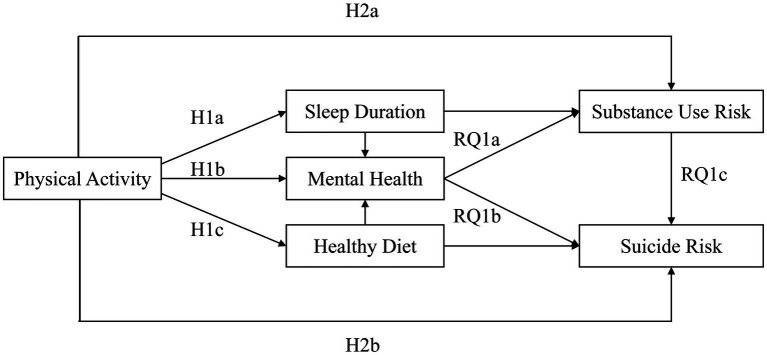
Hypothesized model.

Chain mediation (also called serial mediation) models posit that an independent variable influences the outcome through a sequence of mediators, implying a causal chain among the mediators themselves (43). This model allows researchers to test more complex mechanisms of indirect effects beyond simple mediation (44). In this study, we aim to elucidate the complex mediating mechanisms underlying the relationship among the key variables in the model. In addition, this study seeks to further identify the sequential mediation pathways that explain how these variables are interrelated. We propose the following research question, and present a hypothesized model (Figure 1).

RQ1a: PA is hypothesized to influence SUR through a sequential mediation pathway involving SD and MH.

RQ1b: PA is hypothesized to influence SR through a sequential mediation pathway involving HD and MH.

RQ1c: PA is hypothesized to influence SR through a sequential mediation pathway involving MH and SUR.

## 2 Methods

### 2.1 Data source and participants

The data for this study were derived from the Youth Risk Behavior Surveillance System (YRBSS), a national survey conducted in the United States. The YRBSS was established in 1991 by the Centers for Disease Control and Prevention (CDC) to investigate critical health-risk behaviors among high school students. For the present analysis, we utilized the most recent cross-sectional dataset, collected in 2023.

Institutional review boards at CDC and ICF, the survey contractor, approved the study protocol for YRBSS. All procedures were conducted in accordance with the relevant ethical guidelines and federal regulations. Data collection was carried out in compliance with applicable U.S. federal law and CDC policy. All participants provided voluntary, anonymous responses, and parental consent procedures were followed prior to data collection. The publicly available dataset can be freely accessed from the official CDC website.

Due to the questionnaire targeting to collect disparate information from adolescents, it is not suitable to construct potential variable. However, a recent test–retest study of most of the 2023 survey questions indicated substantial reliability among the questions (45). Although the official guidelines indicate that the high dataset has high reliability, we conducted additional reliability tests across multiple dimensions. The results demonstrated acceptable reliability, as reported in the following sections.

**Figure 2:**
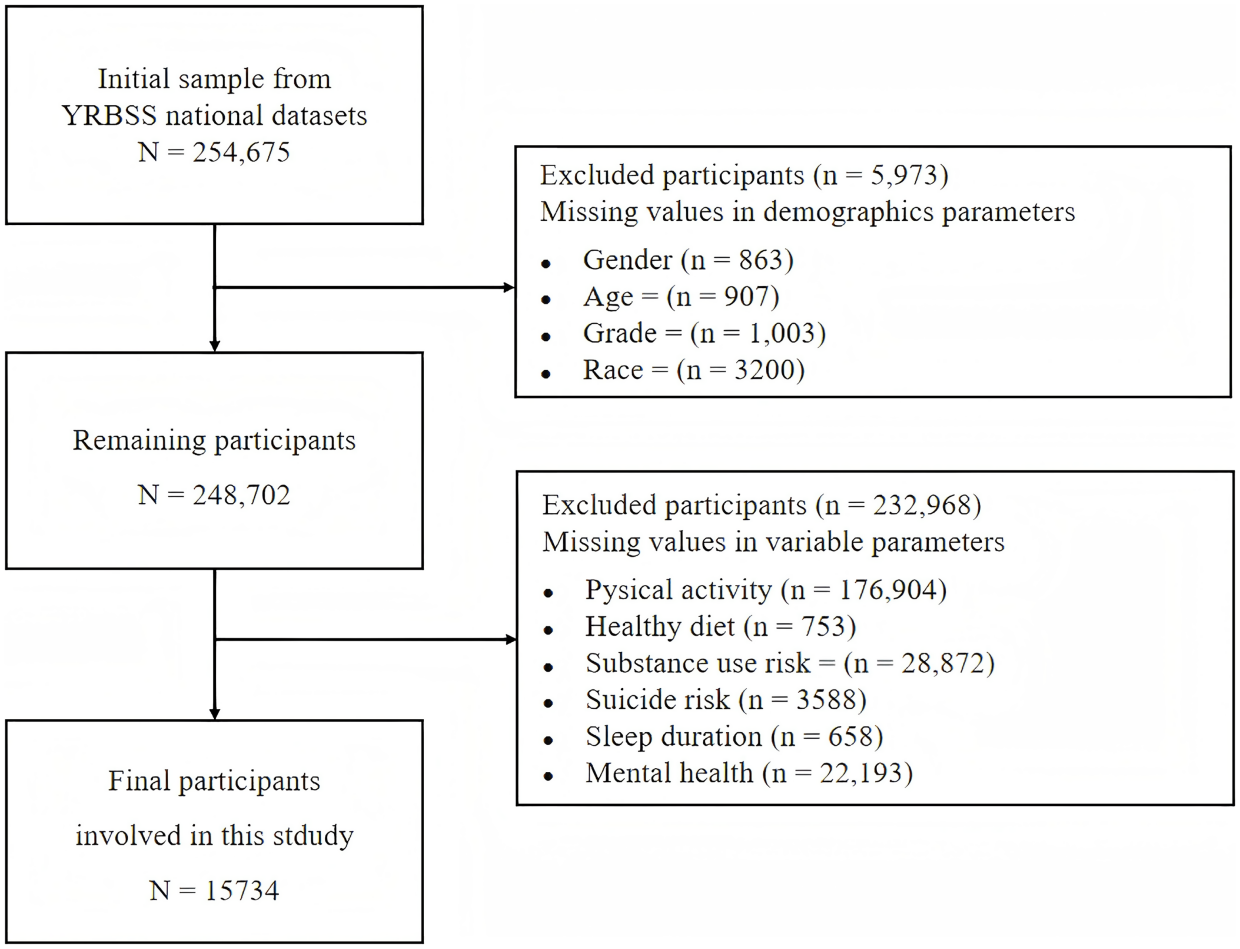
Participant selection flowchart.

The 2023 TRBSS dataset included 254,675 respondents. Figure 2 illustrates the sample filtering process. Participants with missing values in key demographic covariates were excluded, namely gender (*n* = 863), age (*n* = 907), grade (*n* = 1,003), and race (*n* = 3,200), resulting in a remaining sample of 248,702. Subsequently, participants with missing values on core study variables were also excluded. These variables included PA, HD, SUR, SR, SD, and MH. After excluding participants with missing data on any of variables, the final analytic sample comprised 15,734 participants.

### 2.2 Measures

#### 2.2.1 Physical activity

Table 1 presents the variables included in the structural equation model along with their corresponding YRBSS item numbers.

**Table 1:** Variables included in the structural equation model.

Construct	YRBSS item no.	Original question	Response scale	Mean (SD)
Physical activity	Q76	Days physically active ≥60 min/day (past 7 days)	1 = 0 days; … 8 = 7 days	4.97 (2.49)
	Q77	Frequency of take part physical education (PE) classes (in average week)	1 = 0 days; … 6 = 5 days	2.88 (2.17)
	Q78	Number of Sports teams participation (past 12 months)	1 = 0 teams; … 4 = 3 or more teams	1.90 (1.05)
	qmusclestrength	Number of days performing muscle-strengthening exercises (e.g., push-ups, sit-ups, weight lifting) (past 7 days)	1 = 0 days; … 8 = 7 days	3.66 (2.48)
Healthy diet	Q69	Frequency of fruit consumption (past 7 days)	1 = none; 2 = 1 to 3 times; … 7 = 4 or more times per day	2.98 (1.56)
	Q70	Frequency of green salad consumption (past 7 days)	1 = none; 2 = 1 to 3 times; … 7 = 4 or more times per day	1.85 (1.11)
	Q73	Frequency of consumption of other vegetables (excluding green salad, potatoes, and carrots) (past 7 days)	1 = none; 2 = 1 to 3 times; … 7 = 4 or more times per day	2.57 (1.34)
Substance use risk	Q36	Number of days using electronic vapor products (past 30 days)	1 = 0 days; 2 = 1or 2 days; … 7 = all 30 days	1.74 (1.75)
	Q42	Number of days having at least one alcoholic drink (past 30 days)	1 = 0 days; 2 = 1or 2 days; … 7 = all 30 days	1.40 (0.91)
	Q48	Frequency of marijuana use (past 30 days)	1 = 0 times; 2 = 1 or 2 times; … 6 = 40 or more times	1.47 (1.22)
Suicide risk	Q27	Considered attempting suicide (past 12 months)	1 = yes; 2 = no	1.23 (0.42)
	Q28	Made a plan to attempt suicide (past 12 months)	1 = yes; 2 = no	1.18 (0.39)
	Q29	Number of suicide attempts (past 12 months)	1 = 0 times; …5 = 6 or more times	1.16 (0.56)
Sleep duration	Q85	Average number of hours of sleep on school nights	1 = 4 or less hours; 2 = 5 h; … 7 = 10 or more hours	3.44 (1.36)
Mental health	Q26	Felt persistently sad or hopeless for 2 weeks or more, leading to loss of interest in usual activities (past 12 months)	1 = yes; 2 = no	1.43 (0.50)
	Q84	Number of days with poor mental health (e.g., stress, anxiety, depression) (past 30 days)	1 = never; 5 = always	2.79 (1.22)

Items Q26, Q27 and Q28 were reverse-coded such that higher scores represent greater mental health problems.

PA was assessed using four items: frequency of being PA for at least 60 min per day in the past week (Q76), frequency of physical education (PE) class attendance in a typical week (Q77), number of sports teams participated in over the past 12 months (Q78), and frequency of muscle-strengthening activities such as push-ups or weight lifting in the past 7 days (Qmusclestrength). All items were coded so that higher scores reflected higher levels of PA. For example, Q76 ranged from 1 (“0 day”) to 8 (“7 days”), with greater values indicating more frequent PA. These items were treated as indicators of the latent PA construct in the SEM. The internal consistency of the PA construct was acceptable in this study (Cronbach’s *α* = 0.670).

#### 2.2.2 Healthy diet

HD was assessed using three items: frequency of fruit consumption over the past 7 days (Q69), frequency of green salad consumption over the past 7 days (Q70), and frequency of consumption of other vegetables (excluding salad, potatoes, and carrots; Q73). All items were coded such that higher scores indicated higher HD behavior. For instance, Q69 ranged from 1 (“I did not eat fruit during the past 7 days”) to 7 (“4 or more times per day”), with higher values indicating better HD behavior. These items were treated as indicators of the latent HD construct in the SEM. In the present study, the scale demonstrated acceptable internal consistency (Cronbach’s *α* = 0.667).

#### 2.2.3 Substance use risk

SUR was assessed using three items: number of days using electronic vapor products in the past 30 days (Q36), number of days having at least one alcoholic drink in the past 30 days (Q42), frequency of marijuana use (Q48). All items were coded such that higher scores indicated greater SUR. For instance, Q36 ranged from 1 (“0 days”) to 7 (“all 30 days”), with higher values reflecting more frequent use. These items were treated as indicators of a latent SUR construct in the SEM. In the present study, the internal consistency was acceptable (Cronbach’s *α* = 0.738).

#### 2.2.4 Suicide risk

SR was assessed using three items: whether the participant had ever seriously considered attempting suicide in the past 12 months (Q27), whether they had made a plan for attempting suicide in the past 12 months (Q28), and number of suicide attempts in the past 12 months (Q29). All items were coded such that higher scores indicated greater SR. For instance, Q27 was ranged from 1 (“NO”) to 2 (“YES”), with higher values reflecting increased risk. These items were also treated as indicators of a latent SR construct in the SEM. In the present study, the internal consistency of the scale was acceptable (Cronbach’s *α* = 0.931).

#### 2.2.5 Sleep duration

SD was assessed using a single item (Q85): “On an average school night, how many hours of sleep do you get?” Responses were coded such that higher scores reflected longer and healthier SD. The response options ranged from 1 (“4 or less hours”) to 7 (“10 or more hours”), with higher values indicating better SD.

#### 2.2.6 Mental health

MH was assessed using two items. The first item (Q26) asked: “During the past 12 months, did you ever feel so sad or hopeless almost every day for 2 weeks or more in a row that you stopped doing some usual activities?” The second item (Q84) assessed the frequency of poor MH in the past 30 days, defined as experiences of stress, anxiety, or depression: “During the past 30 days, how often was your mental health not good?” Both items were coded such that higher scores indicated poorer MH. For example, Q84 ranged from 1 (“Never”) to 5 (“Always”), with higher values reflecting greater MH problems. These items were treated as indicators of latent MH construct in the SEM.

### 2.3 Statistical analysis

Data processing and statistical analyses were conducted using R 4. 5. For mediation analysis, the bootstrap method was adopted based on Hayes’ recommendation. Compared to Sobel test, the bootstrap method estimates the sampling distribution directly without assuming normality, providing greater statistical power and a lower Type I error rate (46). Arguably, we employed the bootstrap method with 5,000 resamples to estimate the 95% confidence intervals of the mediation effects. All mediation analyses and model fit test were also conducted using R 4.5.

Model fit was considered acceptable when all of the following criteria were met: a non-significant chi-square (*χ*^2^) test result (47). A comparative Fit Index (CFI) and Tucker-Lewis Index (TLI) greater than 0.95. And a Root Mean Square Error of Approximation (RMSEA) being less than 0.08 (48).

## 3 Results

### 3.1 Sample characteristics

Table 2 presents the covariate variables included in this study. Among the final sample, the gender distribution was nearly balanced, with 7,672 females and 8,062 males. Age was categorized into seven groups, ranging from 12 or younger to 18 or older. The distribution approximated normality, with the majority of participants aged 15 (24.9%), 16 (26.0%), and 17 (25.2), which aligns with the typical adolescent age range.

**Table 2:** Demographic characteristics of participant.

Characteristics	Variable (Original codes)	*n*	Percent	Mean (SD)
Gender	Female = 1	7,672	48.8	1.51 (0.50)
	Male = 2	8,062	51.2	
Age (years)	12 or younger = 1	4	0.0	15.95 (1.21)
	13 = 2	19	0.1	
	14 = 3	2072	13.2	
	15 = 4	3,919	24.9	
	16 = 5	4,090	26.0	
	17 = 6	3,962	25.2	
	18 or older = 7	1,668	10.6	
Grade level	9th = 1	3,900	24.8	2.49 (1.11)
	10th = 2	4,081	25.9	
	11th = 3	3,925	24.9	
	12th = 4	3,828	24.3	
	Ungraded or other = 5	0	0	-
Race	White = 1	7,670	48.7	-
	Black or African American = 2	1851	11.8	
	Hispanic/Latino = 3	3,289	20.9	
	All other race = 4	2,924	18.6	

Participants were primarily in grades 9 through 12, with a relatively even distribution across grade levels and a mean grade level of 2.49. Regarding race, White participants comprised the largest group (48.7%), followed by Hispanic/Latino, Black or African American, and other racial categories. We reported frequencies, percentages, and means with standard deviations to provide a comprehensive overview of sample characteristics. All variables included in the correlation matrix were either continuous or ordinal and were treated as approximately continuous.

Table 3 presents the means, standard deviations, and intercorrelations among the key study variables, including gender, age, grade level, PA, HD, SUR, SR, SD and MH. PA was significantly correlated with HD (*r* = 0.26, *p* < 0.001), SUR (*r* = 0.02, *p* < 0.05), SR (*r* = −0.10, *p* < 0.001), SD (*r* = 0.16, *p* < 0.001) and MH (*r* = 0.03, *p* < 0.001). These results indicate that higher levels of PA are associated with higher HD, longer SD, better MH and lower SR. Although a weak positive correlation was observed between PA and SUR, this unexpected association warrants further analysis to clarify the complex relationships among these variables. Race was not included in Table 3, as it is a nominal variable lacking a meaningful numeric scale for correlation analysis. These findings support the hypothesized associations and provide empirical justification for conducting subsequent mediation analysis. While several covariates (e.g., gender, age, grade) showed modest correlations with the main variables, these effects were small and are not considered potential confounders.

**Table 3:** Descriptive statistics and correlation analysis of variable results.

Variable	Mean (S. D.)	1.	2.	3.	4.	5.	6.	7.	8.	9.
1. Gender	1.51 (0.50)	-								
2. Age	15.95 (1.21)	0.04***	-							
3. Grade	2.49 (1.11)	0.01	0.88***	-						
4. PA	3.35 (1.51)	0.24***	−0.12***	−0.15***	-					
5. Health Diet	2.47 (1.05)	0.02*	−0.01	−0.02**	0.26***	-				
6. Substance Use Risk	1.53 (1.08)	0.05***	−0.16***	−0.15***	0.02*	−0.03**	-			
7. Suicide Risk	1.58 (0.21)	−0.17***	−0.01	−0.02**	−0.10***	0.00	−0.30***	-		
8. Sleep Duration	3.44 (1.36)	0.04***	−0.09***	−0.10***	0.16***	0.09***	0.15***	−0.22***	-	
9. Mental Health	2.11 (0.77)	0.06***	−0.01	−0.01	0.03***	0.01	−0.04***	0.05***	0.02*	-

* *p* < 0.05, ** *p* < 0 0.01, ***p < 0.001.

Gender, age, grade, race were categorical variables codes as: Gender (1 = female, 2 = male); Age (1 = 12 or younger, 2 = 13, 3 = 14, 4 = 15, 5 = 16, 6 = 17, 7 = 18 or older); Grade level (1 = 9th grade, 2 = 10th grade, 3 = 11th grade, 4 = 12th grade, 5 = ungraded or other).

### 3.2 Measurement model evaluation

The YRBSS is designed as a surveillance tool that provides observed behavioral indicators rather than latent constructs, and it does not require factor validation such as AVE or CR (49). However, in order to increase the measuring accuracy, we still tried to construct a latent variable (e.g., mental health, substance use risk) in line with prior SEM research and to enhance construct validity.

The measurement model demonstrated acceptable reliability and convergent validity. Standardized factor loadings ranged from 0.34 to 0.84, with most values above the recommended 0.50 threshold (50). Composite reliability (CR) ranged from 0.675 to 0.795, and AVE ranged from 0.401 to 0.568. Although AVE values for PA and Healthy Diet were slightly below the 0.50 criterion, their CR values remained above 0.60, supporting convergent validity (51). Overall, the results confirmed that the latent constructs were measured reliably (Table 4).

**Table 4:** Factor loadings, CR, and AVE of latent variables.

Latent variable	Item	Std. loading	SE	*t*-value	CR	AVE
Physical activity	Q76	0.791	0.020	97.093	0.709	0.401
	Q77	0.344	0.019	39.630		
	Q78	0.497	0.009	59.252		
	QMUSCLE	0.783	0.020	96.136		
Healthy diet	Q69	0.680	0.015	72.880	0.675	0.410
	Q70	0.586	0.010	64.241		
	Q73	0.652	0.012	70.427		
Substance use risk	Q36	0.840	0.014	104.415	0.772	0.534
	Q42	0.624	0.007	76.951		
	Q48	0.713	0.010	88.485		
Suicide risk	Q27	0.839	0.003	115.740	0.795	0.568
	Q28	0.795	0.003	108.202		
	Q29	0.607	0.004	77.549		
Mental health	Q26	0.775	0.004	93.675	0.711	0.552
	Q84	0.710	0.010	86.395		

All factor loadings are significant at *p* < 0.001.

CR, Composite Reliability; AVE, Average Variance Extracted.

### 3.3 Model fit

We used maximum likelihood estimation with robust standard errors (MLR) to handle non-normality. All items were retained without parceling to avoid information loss. Model modifications were not applied to maintain theoretical purity. The model demonstrated the overall model acceptable fit: χ^2^ (93) = 1206.60, *p* < 0.001, RMSEA = 0.0028, 95% CI [0.026, 0.031], CFI = 0.981, TLI = 0.975, SRMR = 0.023. Although the chi-square statistic was significant, which is common in models with large sample sizes and complex structure, alternative fit indices fell within acceptable ranges. The alternative fit indices fell within conventionally accepted thresholds, indicating satisfactory model fit and construct validity.

### 3.4 Structural model

Table 5 presents the standardized path coefficients, standard errors, 95% confidence intervals, and significant levels for the direct effects among the key study variables. PA demonstrated significant direct associations with SR, MH, SD, SUR, HD, with all *p-*values less than 0.001. Specifically, PA significantly predicted better MH (*β* = −0.241, 95% CI [0.036, 0.077]), longer SD (*β* = 0.177, 95% CI [0.159, 0.194]) and higher HD (*β* = 0.395, 95% CI [0.375, 0.415]).

**Table 5:** Standardized direct effects among key study variables.

Path	*β* (Standardized)	SE	95% CI	*p*-value
PA → Suicide Risk	0.047	0.010	[0.036, 0.077]	< 0.001
PA → Mental Health	−0.241	0.012	[−0.263, −0.218]	< 0.001
PA → Sleep duration	0.177	0.009	[0.159, 0.194]	< 0.001
PA → Substance Use Risk	0.085	0.011	[0.064, 0.106]	< 0.001
PA → Healthy Diet	0.395	0.010	[0.375, 0.415]	< 0.001
Mental Health → Suicide Risk	0.739	0.008	[0.724, 0.754]	< 0.001
Mental Health → Substance Use Risk	0.348	0.011	[0.326, 0.371]	< 0.001
Sleep Duration → Mental Health	−0.303	0.009	[−0.321, −0.286]	< 0.001
Sleep Duration → Substance Use Risk (H?)	−0.064	0.010	[−0.084, −0.045]	< 0.001
Healthy Diet → Mental Health	0.059	0.013	[0.033, 0.084]	< 0.001
Heath Diet → Suicide Risk	0.016	0.011	[−0.005, 0.037]	0.130
Substance Use Risk → Suicide Risk	0.114	0.012	[0.091, 0.137]	< 0.001

We observed that two the standardized direct paths showed counterintuitive directions: PA significantly predicted SR (*β* = 0.047, 95% CI [0.036, 0.077]) and SUR (β = 0.085, 95% CI [0.064, 0.106]). Although the direct effect of PA on SR was small and positive, the strong indirect pathway through improved mental health (*β* = −0.241) and its substantial predictive power on suicide risk (*β* = 0.739) suggest that the total effect is largely driven by the indirect mechanisms. The pattern is commonly observed in chain mediation models, particularly when suppressor effects or competing pathways exist. A similar explanation may apply to the relationship between PA and SR.

MH was positively associated with both SR (*β* = 0.395, 95% CI [0.724, 0.754], *p* < 0.001) and SUR (*β* = 0.348, 95% CI [0.326, 0.371], *p* < 0.001). In addition, SD significantly predicted both better MH (*β* = −0.303, 95% CI [−0.321, −0.286], *p* < 0.001) and lower SUR (*β* = −0.064, 95% CI [−0.084, −0.045], *p* < 0.001). HD was positively associated with MH (*β* = 0.059, 95% CI [0.033, 0.084], *p* < 0.001). SUR was a significant positive predictor to SR (*β* = 0.114, 95% CI [0.091, 0.137], *p* < 0.001). However, the direct path from HD to SR was not statistically significant (*β* = 0.016, 95% CI [−0.005, 0.037], *p* = > 0.05). Nevertheless, this path was retained in the mediation analysis, as on indirect effects can still be significant even when the constituent path is not significant (44).

These results suggest that the effect of PA on SR and SUR may operate primarily through indirect pathways (e.g., via MH, SD, HD), providing initial support for the hypothesized mediation structure to be tested in the subsequent analysis.

Table 6 presents the standardized direct, indirect, and total effects from PA to SUR. We observed that the total effect (*β* = −0.021, 95% CI [−0.037, −0.006], *p* < 0.001) shown that higher levels of PA can significantly predict lower SUR, supporting H2a.

**Table 6:** Mediation effects of PA on SUR.

Path	Effect size (β)	SE	95% CI	*p*-value
PA → Mental Health → Substance Use Risk	−0.062	0.004	[−0.070, −0.055]	< 0.001
PA → Sleep Duration → Substance Use Risk	−0.009	0.001	[−0.012, −0.006]	< 0.001
PA → Sleep Duration → Mental Health → Substance Use Risk (H?)	−0.014	0.001	[−0.016, −0.012]	< 0.001
Total Indirect Effect	−0.085	0.005	[−0.093, −0.077]	< 0.001
Direct Effect	0.064	0.008	[0.047, 0.079]	< 0.001
Total Effect	−0.021	0.008	[−0.037, −0.006]	0.008

The mediation analysis revealed several significant pathways. RQ1a (PA → MH → SUR) was supported, indicating that MH significantly mediated the effect of PA on SUR (*β* = −0.062, 95% CI [−0.070, −0.055], *p* < 0.001). More importantly, the pathway (PA → SD → SUR) was also supported, demonstrating that SD significantly mediated the relationship between PA and SUR (*β* = −0.009, 95% CI [−0.012, −0.006], *p* < 0.001). In addition, a significant chain mediation pathway (PA → SD → MH → SUR) was observed (*β* = −0.014, 95% CI [−0.016, −0.012], *p* < 0.001), highlighting a complex multi-step mechanism through which PA influences SUR. These findings suggest that lower levels of PA may initially lead to deteriorated MH or reduced SD, which in turn contribute to increased SUR. The Chain mediation model illustrates that insufficient PA may negatively impact SD, which subsequently impairs MH, ultimately leading to elevated levels of SUR. Together, these results emphasize that both MH and SD are the critical mediating mechanisms linking PA to SUR among adolescents.

Table 7 presents the standardized direct, indirect, and total effects from PA to SR. The total effect (*β* = −0.024, 95% CI [−0.028, −0.021], *p* < 0.001) demonstrates that higher levels of PA significantly predict lower levels of SR, thereby supporting H2b.

**Table 7:** Mediation effects of PA on SR.

Path	Effect size (*β*)	SE	95% CI	*p*-value
PA → Mental Health → Suicide Risk	−0.03	0.002	[−0.033, −0.027]	< 0.001
PA → Healthy Diet → Suicide Risk	0.001	0.001	[0.000, 0.003]	0.117
PA → Substance Use Risk → Suicide Risk	0.002	0.0	[0.001, 0.002]	< 0.001
PA → Mental Health → Substance Use Risk → Suicide Risk	−0.002	0.0	[−0.002, −0.001]	< 0.001
PA → Healthy Diet → Mental Health → Suicide Risk	0.003	0.001	[0.002, 0.004]	< 0.001
PA → Sleep Duration → Mental Health → Suicide Risk	−0.007	−0.007	[−0.007, −0.006]	< 0.001
PA → Sleep Duration → Substance Use Risk → Suicide Risk	0.000	0.0	[0.000, 0.000]	< 0.001
Total Indirect Effect	−0.033	0.002	[−0.037, −0.030]	< 0.001
Direct Effect	0.008	0.002	[0.005, 0.012]	< 0.001
Total Effect	−0.024	0.002	[−0.028, −0.021]	< 0.001

**Figure 3:**
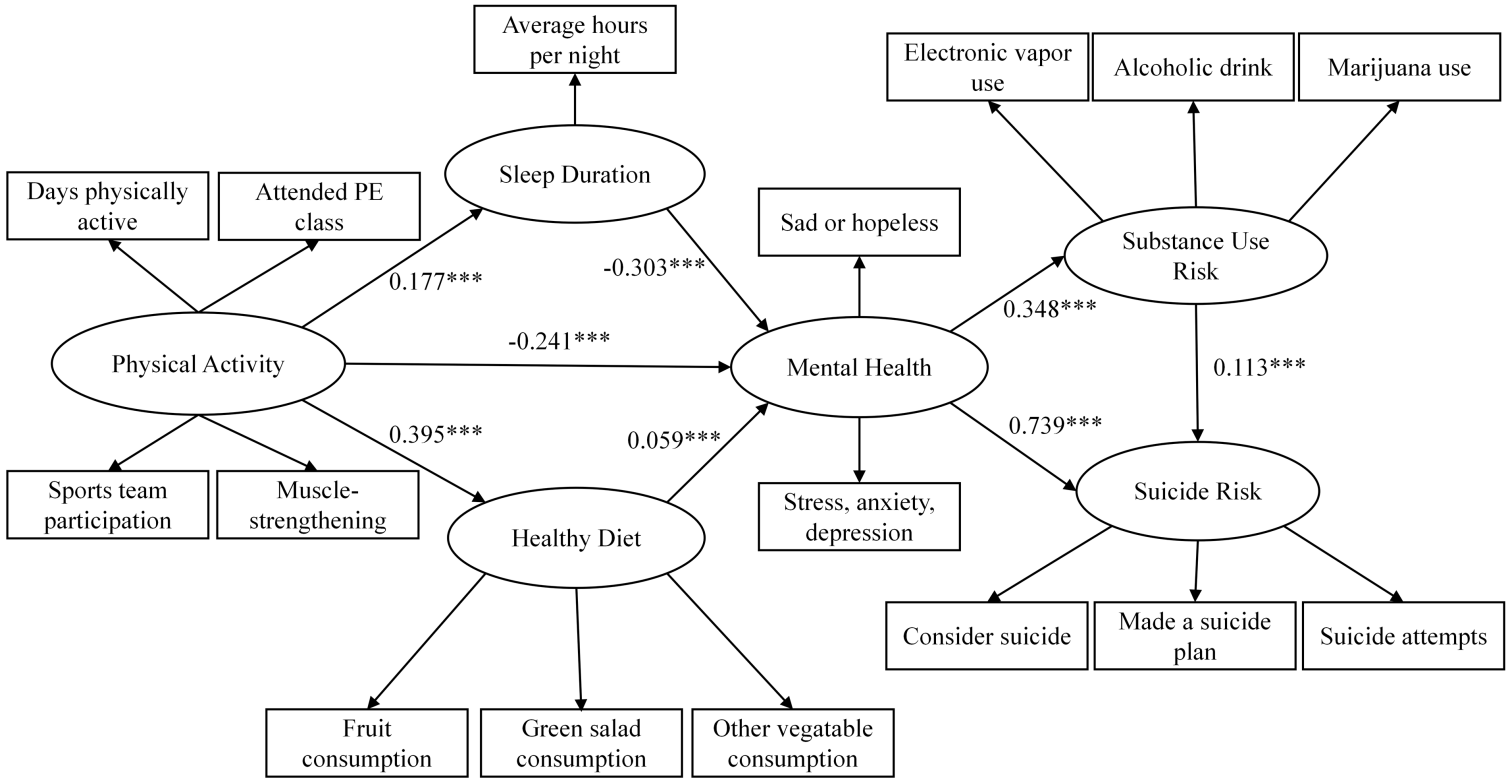
Final structural model with standardized path coefficients.

The mediation and chain mediation analyses revealed several key pathways linking PA to SR. RQ1b was supported, indicating that MH significantly mediated the effect of PA on SR (*β* = −0.03, 95% CI [−0.033, −0.027], *p* < 0.001). This suggesting that higher levels of PA may enhance MH, thereby reducing SR. The pathway from PA to SR visa HD was not supported (*β* = 0.001, 95% CI [0.000, 0.003], *p* < 0.001), indicating that HD did not serve as a significant mediator. However, the indirect effect via SUR was significant (*β* = 0.002, 95% CI [0.001, 0.002], *p* < 0.001), suggesting that PA may reduce SUR, which in turn decreases SR. A significant chain mediation pathway (PA → MH → SUR → SR) was also identified (*β* = −0.002, 95% CI [−0.002, −0.001], *p* < 0.001), indicating that higher levels of PA can improve MH, which subsequently lowers SUR, ultimately leading to minimize SR. Another chain pathway (PA → HD → MH → SR) was supported (*β* = 0.003, 95% CI [0.002, 0.004], *p* < 0.001), highlighting the combined mediating role of HD and MH. Similarly, the sequential pathway (PA → SD → MH → SR) was also significant (*β* = −0.007, 95% CI [−0.007, −0.006], *p* < 0.001), suggesting that PA promotes SD, which enhances MH and consequently reduces SR. In contrast, the pathway (PA → SD → SUR → SR) although statistically significant (*p* < 0.001), yielded a near-zero effect size (*β* ≈ 0.000 95% CI [0.000, 0.000], *p* < 0.001), indicating a lack of practical significance (Figure 3).

## 4 Discussion

### 4.1 Summary of key findings

We examined the direct and indirect relationships between PA, SR, SUR among adolescents, focusing specifically on the mediating roles of SD, HD, and MH. The results generally supported the proposed mediation and chain mediation hypotheses, though with some notable exceptions.

Consistent with hypotheses, higher levels of PA significantly predicted lower overall levels of both SUR and SR. However, direct pathways from PA to SR and SUR were unexpectedly positive, suggesting the presence of suppressor effects and highlighting the critical role of indirect pathways. Indeed, MH emerged as a central mediator, significantly mediating the relationships between PA and both SUR and SR. Additionally, SD was identified as a significant mediator linking PA to SUR, indicating that adequate sleep contributes to reduced substance use risk.

Chain mediation analyses further underscored the complexity of these relationships. PA influenced SUR through a sequential mediation pathway involving SD and MH, highlighting a stepwise protective mechanism (PA → SD → MH → SUR). Similarly, PA influenced SR through multiple significant sequential mediation pathways, including PA → MH → SUR → SR, and PA → SD → MH → SR, reinforcing the central role of MH as a pivotal mediating factor. The sequential mediation pathway via HD and MH (PA → HD → MH → SR) was statistically significant but presented a minor positive effect. Conversely, the sequential mediation via SD and SUR (PA → SD → SUR → SR) were not supported.

These findings illustrate that the beneficial impacts of PA on SR and SUR among adolescents predominantly operate indirectly through improved MH, increased SD, and partially via HD practices. These mechanisms suggest targeted avenues for intervention strategies designed to enhance adolescent risk.

### 4.2 Interpretation of individual hypotheses

Hypothesis H2a was supported, as higher levels of PA significantly predicted lower SUR (*β* = −0.021, *p* < 0.001). This finding aligns with previous studies suggesting physical activity’s protective effects against substance use, possibly through behavioral regulation and healthy lifestyle promotion. For instance, a longitudinal study of adolescents demonstrated that higher engagement in regular PA was associated with reduced likelihood of substance abuse behaviors, supporting our observed protective relationship (52).

Hypothesis H2b was also confirmed; PA significantly predicted reduced SR (*β* = −0.024, *p* < 0.001). Although the direct pathway between PA and SR was unexpectedly small and positive (*β* = 0.047, *p* < 0.001), the robust negative indirect effects primarily via MH indicate a suppression effect. Previous research supports this indirect protective pathway, emphasizing that PA reduces depression and anxiety, thereby lowering SR (53).

Regarding mediation hypotheses, H1a was supported as SD significantly mediated the relationship between PA and SUR (*β* = −0.009, *p* < 0.001). Adequate sleep has been consistently documented as an essential protective factor against adolescent substance use, likely by promoting emotional regulation and reducing impulsivity (54).

Hypothesis H1b was strongly supported, highlighting MH as a significant mediator in the relationships among PA, SUR, and SR (*β* ranging from −0.03 to −0.062, *p* < 0.001). Consistent with earlier findings, our results underline MH as a central explanatory factor linking physical activity to adolescent mental health outcomes, including SUR (55) and SR (56).

Hypothesis H1c regarding HD mediating effect of PA on SR was not supported (*β* = 0.001, *p* < 0.001). Although HD was positively associated with improved MH, the direct mediating role of HD alone was negligible. This outcome aligns with studies suggesting dietary effects on mental health are subtle and often operate indirectly via complex biopsychosocial pathways rather than simple, direct mediation (57).

Chain mediation hypotheses (RQ1a) received support. Revealing a significant sequential pathway from PA through SD and MH to SUR (β = −0.014, p < 0.001), suggesting a cumulative protective process through improved sleep and subsequent mental health enhancement. This finding is congruent with existing literature emphasizing multi-step health behavior chains among adolescents (58).

RQ1b was also confirmed, demonstrating significant chain mediation through HD and MH to SR (*β* = −0.007, *p* < 0.001). This result emphasizes the complex interplay among behavioral health factors in reducing suicide risk, consistent with the previous theoretical model proposed (59).

RQ1c, involving chain mediation via MH and SUR (*β* = −0.002, *p* < 0.001), was statistically significant, suggesting that complex interplay among mental health factors in reducing suicide risk. This finding is congruent with existing literature emphasizing multi-step health behavior chains among adolescents (60).

These interpretations underline the central role of indirect pathways—particularly mental health—as the primary mechanisms through which physical activity influences critical adolescent health outcomes.

### 4.3 Interpretation of mediation effects

The mediation analyses provide robust support for indirect pathways linking PA to SUR and SR, highlighting the critical roles of MH and SD. MH was the most influential mediator, significantly mediating relationships between PA and both SUR and SR. Adolescents with higher PA typically experience improved mental health, consequently reducing SUR and SR, consistent with prior findings emphasizing MH as a key intervention target. SD also significantly mediated the relationship between PA and SUR, reinforcing prior research that adequate sleep contributes to better emotional regulation and reduced substance use among adolescents.

However, the mediating role of HD between PA and SR was negligible, suggesting dietary influences alone may have limited practical significance. Chain mediation analyses further supported multi-step pathways (PA → SD → MH → SUR; PA → MH → SUR → SR), underscoring the interconnectedness of these health behaviors in reducing risk.

### 4.4 Theoretical contributions

The results indicated several important theoretical contributions to the literature on adolescent health behaviors and risk prevention. It enriches the understanding of the mechanisms linking PA to SR and SUR by highlighting the central mediating roles of MH and SD. The identification of MH as a pivotal mediator underscores its significance within adolescent behavioral health models, reinforcing theories that emphasize emotional and psychological regulation as critical pathways through which lifestyle behaviors impact health outcomes.

By explicitly examining sequential mediation (chain mediation) pathways involving SD, MH, and SUR, this study advances current conceptualizations of health behavior interconnectivity among adolescents. It demonstrates empirically that PA exerts its protective effects through complex, multi-step processes, emphasizing an integrated approach to health promotion rather than isolated behavioral interventions.

In conclusion, the complex positive direct associations observed between PA and both SR and SUR contribute to the theoretical discourse on suppressor effects and competing mechanisms in behavioral health research. These findings highlight the necessity of considering indirect and suppressor effects within health behavior theories and interventions, urging related practitioners to adopt a nuanced understanding of how protective behaviors may sometimes interact with contextual factors in complex ways.

### 4.5 Practical implications

The findings provide important practical implications for practitioners, educators, and policymakers aiming to promote adolescent well-being. The identification of MH and SD as key mediators between PA and adolescent risk behaviors underscores specific target areas for intervention programs.

Interventions designed to enhance MH among adolescents through regular PA should be prioritized. The related institutions could implement structured PA integrated with emotional and psychological support to foster comprehensive health benefits. Additionally, given SD protective role against SUR, educational programs emphasizing healthy sleep hygiene and routines may effectively complement existing SUR prevention efforts.

Moreover, recognizing the chain mediation effect (PA → SD → MH → SUR) highlights the value of adopting integrated intervention strategies. Practitioners should consider multi-component programs that simultaneously address PA levels, SD, and MH to optimize reductions in SUR and SR among adolescents.

### 4.6 Limitations

Despite its contributions, the current study has several limitations. First, the cross-sectional design prevents causal inferences from being established, as it does not allow for the determination of temporal sequence among variables. And family environment factors (e.g., parental supervision, economic status) and social support (e.g., partner relationship) may simultaneously influence PA, SD, MH, and SUR. Longitudinal or experimental designs are required to verify the directionality and causal relationships identified in the model. Second, the generalizability of our findings is limited by the sample composition, which included only U.S. adolescents. The lack of data from developing countries and some minority groups may restrict the universality of the conclusions. Future studies should validate these relationships in more diverse, multi-ethnic, and cross-national samples to enhance external validity. Third, the YRBSS does not collect detailed physical activity dose metrics, such as total weekly energy expenditure (e.g., MET-min/week), which limits our ability to examine potential dose–response or threshold effects, including the possibility that excessive activity could have adverse impacts on sleep or mental health. Finally, employing the identified model as a basis for designing and implementing longitudinal intervention studies could yield more definitive insights into the pathways connecting physical activity and adolescent health outcomes.

## 5 Conclusion

(1) PA significantly reduced SR and SUR, primarily through improved MH and increased SD, highlighting these factors as critical mediators. (2) Although HD alone did not mediate the relationship between PA and SR, it showed modest effects in sequential mediation pathways. (3) Significant chain mediation pathways were identified, demonstrating that PA enhances sleep duration, which subsequently improves mental health, ultimately reducing SUR and SR.

These findings emphasize that MH and SD play crucial indirect roles in linking PA with adolescent health outcomes, suggesting integrated strategies promoting PA, MH support, and sleep quality as effective approaches for reducing SR and SUR among adolescents. Although our study does not directly evaluate intervention programs, these results provide a theoretical basis for developing and testing multi-component approaches in future experimental or longitudinal research.

## Data Availability

Publicly available datasets were analyzed in this study. This data can be found here: https://www.cdc.gov/yrbs/about/index.html.
